# Tailored Functionalization of Plasmonic AgNPs/C:H:N:O Nanocomposite for Sensitive and Selective Detection

**DOI:** 10.1002/jbio.202400353

**Published:** 2024-12-23

**Authors:** Sanjay Kumar, Hana Maskova, Anna Kuzminova, Paval Curda, Lenka Doudova, Jan Sterba, Ondřej Kylián, Ryan O. M. Rego, Vítězslav Straňák

**Affiliations:** ^1^ Faculty of Science University of South Bohemia Ceske Budejovice Czech Republic; ^2^ Biology Centre ASCR Institute of Parasitology Ceske Budejovice Czech Republic; ^3^ Faculty of Mathematics and Physics Charles University Prague Czech Republic

**Keywords:** ag nanoparticles, borrelia, C:H:N:O thin film, localized surface plasmon resonance, Lyme disease, magnetron sputtering, nanocomposite, nylon, plasma polymer, surface functionalization

## Abstract

We report here on the development of tailored plasmonic AgNPs/C:H:N:O plasma polymer nanocomposites for the detection of the pathogenic bacterium *Borrelia afzelii* , with high selectivity and sensitivity. Silver (Ag) nanoparticles, generated by a gas aggregation source, are incorporated onto a C:H:N:O plasma polymer matrix, which is deposited by magnetron sputtering of a nylon 6.6. These anchored Ag nanoparticles propagate localized surface plasmon resonance (LSPR), optically responding to changes caused by immobilized pathogens near the nanoparticles. The tailored functionalization of AgNPs/C:H:N:O nanocomposite surface allows both high selectivity for the pathogen and high sensitivity with an LSPR red‐shift Δλ > (4.20 ± 0.71) nm for 50 Borrelia per area 0.785 cm^2^. The results confirmed the ability of LSPR modulation for the rapid and early detection of (not only) tested pathogens.

## Graphical abstract

AgNPs/C:H:N:O nanocomposite as a highly selective and sensitive transducer for detecting Borrelia bacteria was developed. The functionalized surface enables precise immobilization of spirochetes, with a measurable LSPR red‐shift indicating pathogen presence. Demonstrating a detection limit of 50 Borrelia per 0.785 cm^2^, the system offers rapid, cost‐effective, and practical point‐of‐care diagnostic potential for Lyme disease and other infectious pathogens.

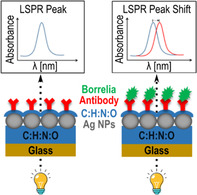

**Abbreviations**
AgNPs: silver nanoparticles
FM: fluorescence microscopy
FMHM: full width at half maximum
GAS: gas aggregation nanoparticle source
LD: lyme disease
LSPR: localized surface plasmon resonance
NPs: nanoparticles
PBS: phosphate‐buffered saline
SEM: scanning electron microscopy
TBS: tris‐buffered saline

## 1 Introduction

Sensitive and selective detection of specific factors within biological systems is of high interest for early diagnostics in biomedicine. Strategies utilizing optical and photonics approaches offer great promise in detection, imaging, visualization in‐time monitoring, etc., making it important for the development of advanced diagnostic tools [1, 2, 3]. Localized Surface Plasmon Resonance (LSPR) [4, 5] on nanoparticles made of plasmonic materials belongs to such widely used techniques.

LSPR is an optical phenomenon that occurs when light interacts with metal nanoparticles whose size is smaller than the wavelength of the incident light [6, 7, 8, 9]. The plasmonic properties of metal nanoparticles do not depend only on their material, size, shape, or interparticle distance but also on the surrounding medium (its permittivity) [10, 11, 12, 13]. The modulation of the LSPR curve as a response to surface property changes caused by the immobilization of the target agent (bacteria) makes the LSPR suitable for (bio)detection. Over the past decade, the LSPR concept was successfully used to detect bio‐substances [14, 15, 16, 17, 18, 19] such as proteins, viruses [20, 21, 22, 23, 24, 25], molecules [26, 27, 28, 29, 30], etc. The most significant parameter is the LSPR peak shift Δλ, which is monitored by responding to surface modification [31, 32]. The principle of LSPR is also utilized in advanced sensing techniques such as Surface‐Enhanced Raman Spectroscopy (SERS) [33], where a nanostructured surface provides a significant increase in electric field intensity. Recently, an enhanced variation known as electrically modulated SERS (E‐SERS) has been reported [34, 35], which further strengthens the signal by applying an external electric field.

In this work, we introduce a successful concept for fast, selective, and sensitive LSPR plasmonic detection of the Lyme Disease (LD) bacterial pathogen. It is extremely beneficial in medical/diagnostic fields if the pathogen causing the disease, in this case, the LD bacterium, is detected in the blood after infection so that antibiotic treatment can be provided early rather than waiting for the production of antibodies in patients and serological testing. Many cases are misdiagnosed or not diagnosed due to non‐specific symptoms and inadequate testing [36]. Early diagnosis is crucial for efficient antibiotic treatment to suppress the development of acute illness; if antibiotic treatment is delayed, it may not be effective [37]. Current diagnostics primarily rely on molecular biology, serological, or PCR techniques, which are expensive, complicated, and typically provide the result with delay [38, 39]. For that reason, quick, highly selective, sufficiently sensitive, easy to operate, and cheap sensors for Borrelia detection are needed.

The proposed concept is based on the LSPR effect of functionalized nanocomposite of plasma‐polymerized C:H:N:O film with embedded Ag nanoparticles for fast, selective, and sensitive detection of the Borrelia pathogen. Plasma polymer films are polymeric materials with an irregular structure and a high degree of cross‐linking and branching prepared by plasma‐assisted deposition [40, 41]. The variety of structures results from the formation of free radicals in the plasma volume and subsequent random polymerization of monomer units onto the surface. The plasma polymer films have disposing properties that are appreciated in many applications [42, 43]. One advantage of plasma polymer thin films is the presence of various functional groups, either derived from the precursor molecules or inherent in their polymerizing units. These films can retain high concentrations of specific groups such as OH [44], —NH_2_ [45, 46], or —COOH [47], as well as other groups like —COOR, —Br, —SH, and —CF_x_ [40]. This variety of functional groups provides a wide range of achievable properties [48], which are particularly valuable for the selective immobilization of biomolecules [49, 50] and highly appreciated for creating effective surfaces for sensing [51].

The functionalization, utilizing natural —NH_2_ grafts of plasma polymerized C:H:N:O, in combination with specific antibodies, allows selective detection of Borrelia lysate, and live Borrelia, all from the pathogenic bacterium *Borrelia afzelii* . The detection principle uses the localized surface plasmon resonance effect [6, 7, 8, 9, 52] on Ag nanoparticles prepared by a gas aggregation source (GAS) [53, 54, 55]. The plasmonic properties of these nanoparticles are, among others, strongly influenced by the surrounding medium, particularly its permittivity [8, 10, 11, 12, 13]. For instance, if the surrounding medium is polarized, it decreases the restoring force of the electron cloud, which can shift the LSPR frequency, resulting in a red‐shift of the LSPR absorption in UV–VIS spectra [6]. Hence, the modulation of the LSPR curve reveals specifically immobilized target agents—in our case, Borrelia pathogens.

This work provides a successful concept for fast, selective, and sensitive detection of Borreliosis pathogens. The proposed diagnostic system would allow for the rapid and early detection of the pathogen causing Lyme disease and faster, personalized treatment of the patients.

## 2 Materials and Methods

### 2.1 Fabrication of AgNPs/C:H:N:O Nanocomposite

**FIGURE 1:**
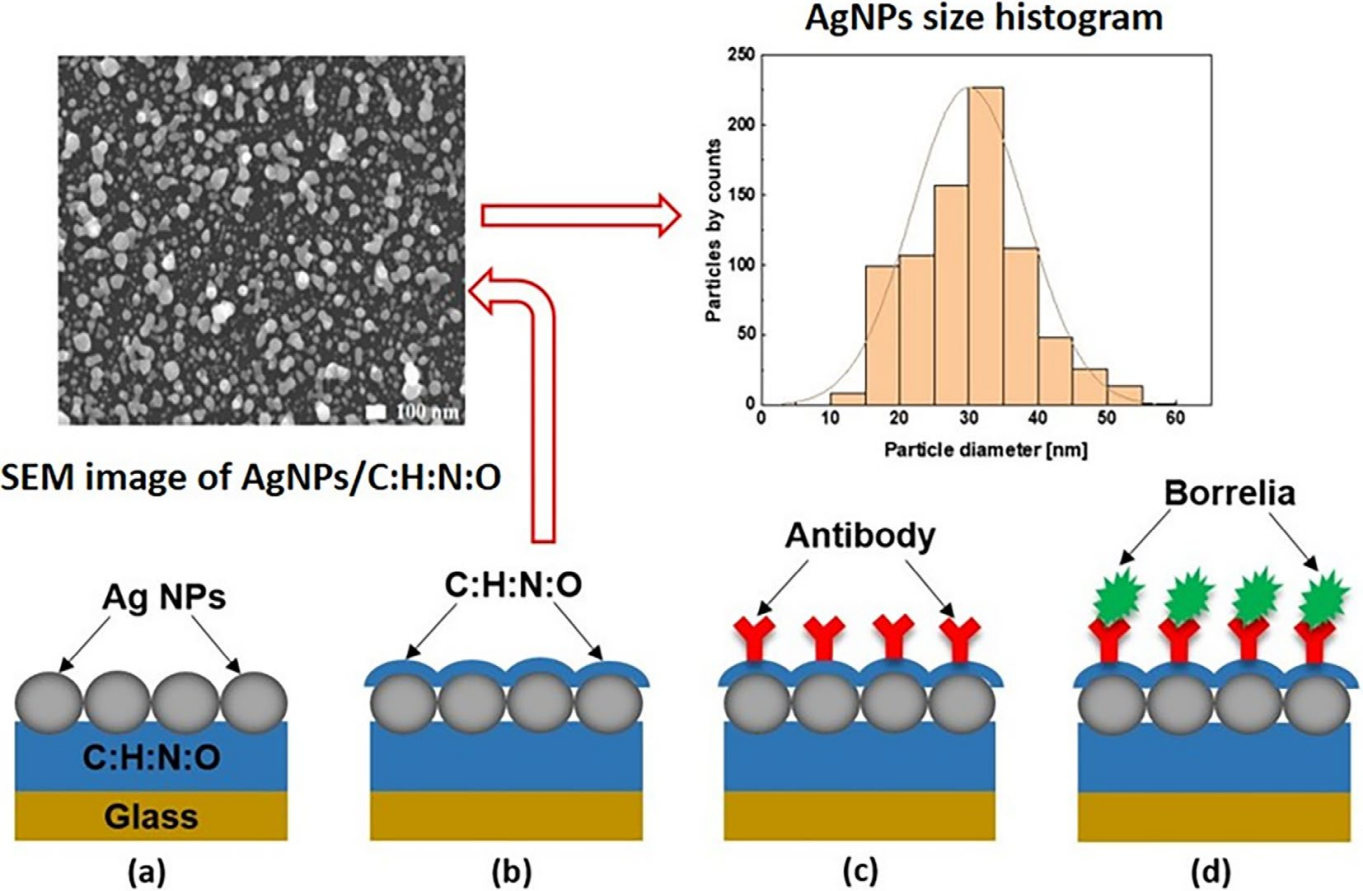
The SEM image of AgNPs/C:H:N:O nanocomposite with 10 nm C:H:N:O film on the top, ready for functionalization. The histogram shows the size distribution of AgNPs of about 30 nm. Schematic sequence of nanocomposite functionalization steps (lower row): (a) AgNPs/C:H:N:O nanocomposite deposited on a glass substrate, (b) nanocomposite with 10 nm of C:H:N:O plasma polymer, (c) surface functionalized with a specific antibody making the surface ready for (d) capture of Borrelia.

The AgNPs/C:H:N:O nanocomposite functions as a transducer for monitoring LSPR signal modulation, with the plasma‐polymerized C:H:N:O matrix stabilizing and anchoring the plasmonic Ag nanoparticles, as shown in Figure 1. The nanocomposite exhibits an irregular and complex morphology [55, 56], with an estimated mean size of NPs (30 ± 5) nm, characterized by the log‐normal histogram distribution [57] having a surface coverage ∑_NPs_ = 65%.

The nanocomposite was prepared in two steps. First, the plasma‐polymerized C:H:N:O matrix was created by RF magnetron sputtering, using a power of 40 W (Comet citoPlus 1000 W, 13.56 MHz) on a nylon 6.6 target (Goodfellow) in a vacuum chamber at a pressure of 3 Pa, with an Ar/N₂ = 75/25 working atmosphere. For detailed experimental conditions, refer to our previous studies [58, 59]. The C:H:N:O film was deposited over 315 s, resulting in a thickness of 15 nm, measured with a (Tencor Alpha‐Step D‐600) profilometer and independently verified by Ellipsometry (J.A. Woollam M‐2000). A 60 W halogen lamp for in situ heating was used to raise the temperature of the substrate and C:H:N:O film to approximately 150°C during growth.

In the following step, Ag nanoparticles were deposited using a home‐built gas aggregation source (GAS) based on Haberland's design, where a planar magnetron (KJ Lesker) serves as the material source within a water‐cooled aggregation chamber [60]. A 3‐in., 6 mm‐thick Ag target (Goodfellow) was sputtered at a pressure of 40 Pa, using a constant DC current of 0.2 A (Henzinger PNC 1500–800) to produce supersaturated metal vapors that aggregate into nanoparticles (NPs). These NPs then exited the GAS through a 2 mm orifice in the form of a beam. Since diatomic gases enhance NPs deposition speed [61], a reactive gas mixture of Ar/N₂ = 15/5 was used. The distance between the GAS output orifice and the substrate was 25 cm, and the Ag NP deposition time was set to 5 min. The resulting AgNPs/C:H:N:O nanocomposite was deposited onto 10 mm diameter circular silica glass substrates.

The first functionalization step was done by a 10 nm thin layer of plasma polymer C:H:N:O onto the nanocomposite surface (Figure 1b). The film was deposited by RF magnetron sputtering of a nylon 6.6 target in a nitrogen‐containing atmosphere, i.e., a strategy previously shown to allow for the production of thin and compact films rich in primary amino groups [62]. Such functional groups are known to covalently bind various types of biomolecules [17], including antibodies used for biosensing applications [63, 64]. In this study, the magnetron sputtering was performed at 40 W (Comet citoPlus 1000 W, 13.56 MHz) in a reactive atmosphere Ar/N_2_ = 75/25 at the pressure of 3 Pa. We now refer to this prepared surface as a ‘capture slide’ [65].

### 2.2 Target Agent: Bacteria and Lysate Preparation

The *Borrelia afzelii* strain CB43 is a low‐passage infectious clonal isolate [66, 67]. CB43 cultures were grown at 34°C in a liquid BSK‐H medium (Sigma‐Aldrich/MERCK) containing 6% rabbit serum. A 7 mL culture of Borrelia (CB43) was centrifuged (8.000 × g) for 10 min, washed with TBS, resuspended in TBS, and 2.7 × 10^7^ Borrelia per sample was used for the detection of live Borrelia. The first detection of the minimal level of Borrelia detected by the system was performed using the decimal dilution range of Borrelia (50–10.000 Borrelia/sample). After the positive detection of 100 Borrelia/sample (diameter 10 mm, area 0.7854 cm^2^), the experiment was repeated with 50 and 100 Borrelia per sample to reveal the detection limit. To prepare the Borrelia lysate, 7 mL of the Borrelia culture was lysed using 4% sodium dodecyl sulfate in TBS for 15 min, followed by 30 min of sonification. The concentration of proteins measured was at 700 ng/sample.

### 2.3 Functionalization of AgNPs/C:H:N:O Nanocomposite for Target Selective Immobilization

Capture slides were twice washed with phosphate‐buffered saline, pH 7.4 (PBS), and specific antibodies (goat anti‐rabbit HRP or the anti‐DbpA serum) were added for immobilization for 1 h at room temperature [68]. Capture slides were then triple‐washed with Tris‐buffered saline, pH 7.4 with 0.05% Tween‐20 (TBS‐T) followed by immersion in a blocking solution (0.15 M glycine in TBS‐T) for 30 min to block the free protein‐binding functional groups. We will now refer to such a functionalized nanocomposite surface as a “ready‐to‐use” sensor transducer (Figure 1c).

LSPR detection of specifically immobilized targets was carried out using: (i) a positive control: rabbit anti‐dolphin polyclonal antibody, (ii) Borrelia lysate, and (iii) live Borrelia suspension; always incubated onto the “ready‐to‐use” sensor transducer for 1 h at room temperature. After this period, samples were twice washed with TBS‐T and once with Tris‐buffered saline, pH 7.4 (TBS). LSPR detection in fresh TBS was analyzed using UV–VIS spectrophotometry (Shimadzu UV‐1800). The UV–Vis spectra were analyzed in the spectral range from 325 nm to 600 nm with spectral accuracy and reproducibility of 0.1 nm. The recorded values of transmittance for each wavelength were subsequently converted to absorbance. The background of the glass substrate was subtracted from the measured absorbance. In all cases, the value of absorbance was below 1.

Here we used:

1. Specific antibodies: goat anti‐rabbit HRP antibody for primary system optimization with Ab+Ab (1:1000 in PBS‐T; Vector Laboratories, USA), and 6% rabbit serum (1:200 in PBS‐T; anti‐DbpA, own production, concentration of stock 37,25 μg/mL; see Figure S3), anti‐DbpA is LD Borrelia specific [68].
2. Positive control: rabbit anti‐dolphin polyclonal antibody (1:1000 in TBS; Abcam, USA) was used for Ab+Ab optimization.

The attachment of specific anti‐DbpA antibody onto a sensor transducer was detected via indirect immunofluorescence method in comparison to negative control which was non‐specific pre‐immunization rabbit serum. Antibodies (1:200) were attached to the sensor transducer for 1 h at room temperature. After this period, three washes with PBS‐T were performed. Non‐specific bonds were blocked using 0.15 M glycine in PBS‐T for 45 min. Goat anti‐Rabbit IgG H&L (DyLight 488) (DI‐1488, Vector Laboratories) was added (1:400) and incubated for 1 h at room temperature. Samples were washed three times with PBS‐T and allowed to dry. A mounting medium for fluorescence (Vectashield) was applied to the samples and fluorescence signals were observed using Olympus BX53. Moreover, the concentration of the attached antibody was measured using ELISA. Sensor transducers were washed with PBS twice. 100 μL AntiDbpA antibody or negative control serum was added to each sample (1:200) for 1 h at 37°C. Three washing steps with PBS‐T were performed. Samples were blocked with 4% BSA in PBS (200 μL) for 45 min at 37°C followed by three wash steps with PBS‐T. HRP Goat Anti‐Rabbit IgG (H + L) Antibody (Peroxidase) (AS014, Abclonal; 100 μL) was then added to each sample (1:2500) for 1 h at 37°C. Three wash steps with PBS‐T were done. TMB substrate (ES022, Merck, 100 μL) was added to each well and incubated at 37°C. Reactions were stopped after 10 min using 0.3 M sulphuric acid. Absorbances were measured by Synergy H1 microplate reader (Biotek) at 450 nm. Concentrations of the attached antibody onto the surface area were calculated from the calibration curve (Figure S3).

The verification of spirochete attachment onto a “ready‐to‐use” sensor transducer in comparison to the control “buffer + block + positive ctrl” sample was performed using the indirect immunofluorescence method. The beginning of this procedure was the same as described above. Since we used a larger area of samples (19.76 cm^2^), the concentration of spirochetes was increased appropriately. In other words, 2.5 × 10^6^ Borrelia per sample was used instead of 10^5^ Borrelia per sample. After immobilization and washing steps, spirochetes were fixed with Roti‐Histofix 4% (Roth) for 1 hour and washed three times with PBS. Next, samples were treated for 15 min with 0.1% Triton X‐100 in PBS for permeabilization, followed by washing twice with PBS. After that, the formaldehyde autofluorescence was blocked (twice) with 50 mM NH_4_Cl in 1% BSA in PBS for 10 min. Samples were washed after this incubation and the blocking of non‐specific binding was performed using 3% BSA in PBS for 1 h. Anti‐DbpA serum in 3% BSA in PBS (1:200), [68] was used as the specific antibody, and samples were incubated overnight at 4°C. Samples were then washed five times with PBS for 5 min and the specific antibody was labeled with DyLight 488 Goat Rabbit IgG Antibody in 3% BSA in PBS for 1 h (1:400; Vector Laboratories). Finally, samples were washed five times with PBS for 5 min and mounted in Vectashield mounting media (Vector Laboratories). Analysis of Borrelia spirochetes attached to the sensor was performed using an Olympus BX‐53 fluorescence microscope equipped with an Olympus DP‐70 CCD camera (40× magnification).

## 3 Results and Discussion

### 3.1 LSPR Response Caused by the Functionalization of Nanocomposite Surface

**FIGURE 2:**
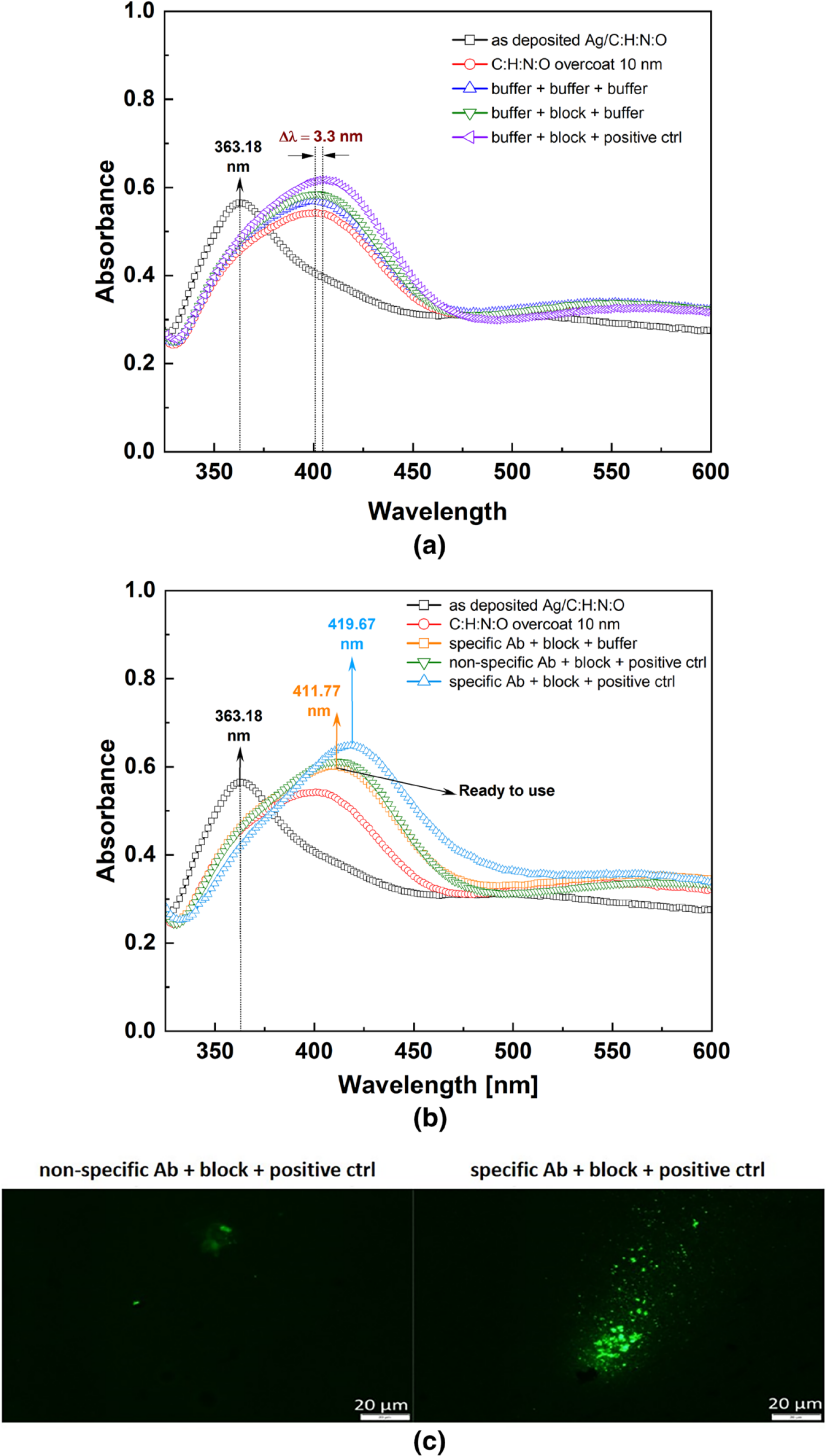
(a) UV–VIS spectra of: (i) as deposited AgNPs/C:H:N:O, (ii) AgNPs/C:H:N:O coated with additional C:H:N:O layer resulting in the spectral shift Δλ_LSPR_ = 36.79 nm, (iii) overcoated AgNPs/C:H:N:O coatings exposed to different control solutions. (b) The LSPR modulation of the UV–VIS absorbance due to specific antibodies and positive control (polyclonal antibodies) grafted onto the surface. The nanocomposite surface with grafted specific Ab and blocked non‐specific binding we referred to as a “ready‐to‐use” transducer surface. (c) The verification of the specific antibody attachment onto the sensor transducer in comparison to non‐specific antibody.

The LSPR curves measured for non‐chemically functionalized surfaces are summarized in Figure 2a and correspond with the measurements specified in Table 1. Deposited bare AgNPs/C:H:N:O nanocomposite provides an LSPR peak with maximum absorption at 363.18 nm, which corresponds with the value reported elsewhere [58, 59]. We also characterized the LSPR peak by its full width at half maximum FMHM = 50.87 nm and the absorbance γ; expressed here with the arbitrary units. Furthermore, Figure 2a displays the LSPR curve measured for the AgNPs/C:H:N:O nanocomposite covered by the 10 nm thick C:H:N:O plasma polymer layer. We can see that the LSPR peak is red‐shifted with the maximum at the wavelength of about λ_LSPR_ = 399.97 nm (the spectral shift Δλ_LSPR_ = 36.79 nm) with significantly broader FWHM = 83.39 nm. This LSPR modulation is caused by the change of the dielectric constant of the Ag environment due to the presence of the uppermost C:H:N:O layer [52]. The absorbance is not significantly influenced by the deposited plasma polymer.

**TABLE 1:** The flow chart of the experiments carried out for verification of the sensor functionality and LSPR modulation. Different approaches of surface functionalization, blocking of non‐specific binding, and detection of various species and their combinations were tested. The details are shown in the Tables S1, S2, respectively, in the supporting information.

No.	Activation	Blocking	Detection	Type of control	Notation
(1)	Buffer	Buffer	Buffer	*Control of surface response*	buffer + buffer + buffer
(2)		Block	Buffer	*Control of blocked surface response* (NEG)	buffer + block + buffer
(3)			Positive control	*Control of blocked surface response* (POS)	buffer + block + positive ctrl
(4)	Specific Antibody	Block	Buffer	*Control of active surface response* (NEG)	specific Ab + block + buffer
(a)	Specific Antibody	Block	Positive control	*Response on detection* (POS) *– polyclonal antibody*	specific Ab + block + positive ctrl
(b)			Lysate	*Response on detection* (POS) *– Borrelia Lysate*	specific Ab + block + lysate
(c)			Borrelia	*Response on detection* (POS) *– Borrelia afzelii CB43*	specific Ab + block + Borrelia

To verify the applicability of functionalized AgNPs/C:H:N:O nanocomposite for LSPR detection, the bio‐receptor selectivity was tested first. LSPR modulation caused by blocking solutions and positive control (polyclonal antibodies) was studied to eliminate any false results. The combination of three buffer solutions PBS‐T, TBS‐T, and TBS was employed (see Table 1) and the resulting LSPR curve was noted as “buffer + buffer + buffer” (Figure 2a). No significant LSPR shift was observed as compared to spectra without buffers. A similar result was reached when the Glycine in TBS‐T was used (see “buffer + block + buffer” curve in Figure 2a). This proved that the solution of 0.15 M Glycine in TBS‐T, which serves as the blocking element for the free protein‐binding functional groups and is used in the middle step between PBS‐T and TBS, does not affect the surface and the LSPR response. The curves “buffer + buffer + buffer” and “buffer + block + buffer” can be thus considered almost identical in the frame of measurement error; see absorbance curves peaking at ~400 nm in the frame of an acceptable error. Since both of these solutions are amino‐graft free (glycine contains one —NH_2_ group in its composition), we attribute slight modulation of the LSPR curve by the molecule mechanical adhesion and immobilization due to weak Van der Waals forces, expecting that the Coulombic and London dispersive forces of water dipoles with C:H:N:O surfaces play a dominant role.

A more pronounced difference is observed for the UV–VIS spectra of the “buffer + block + positive ctrl” sample. In this case, the LSPR peak maximum is at the wavelength λ_LSPR_ = 404.31 nm, which corresponds to the spectral shift difference Δλ_LSPR_ = 3.3 nm as compared to “buffer + buffer + buffer” and “buffer + block + buffer” samples (Figure 2a). This spectral shift indicates at least partial immobilization of the polyclonal Ab (positive control) onto the surface despite the previous blocking of the grafts. The immobilization of the polyclonal Ab also causes a slight enhancement of absorbance.

The modulation of the LSPR curve caused by grafting of the specific goat anti‐rabbit HRP antibody due to —NH_2_ grafts of plasma polymer C:H:N:O is shown in Figure 2b. The specific antibody grafting onto the surface, which happens through the interaction of primary amino groups with the carboxyl‐terminal end of the specific antibody leading to the covalent immobilization of antibodies on amino‐rich surfaces [17], shifts the LSPR peak, reference to “specific Ab + block + buffer” about Δλ_LSPR_ = 11.8 nm, reaching the maximum at 411.77 nm and providing the higher absorbance. Surface grafted with specific antibodies and blocked non‐specific grafts by Glycine in TBS‐T solution we consider as a “ready‐to‐use” transducer surface.

After the blocking procedure, the control rabbit anti‐dolphin polyclonal antibody, which is non‐specific to any Borrelia proteins, when compared to the specific DbpA antibody, was incubated to verify the grafting and detection ability of the proposed LSPR strategy. The positive control (rabbit anti‐dolphin polyclonal antibody), serving at this moment as a target of detection, makes another red‐shift of about Δλ_LSPR_ = 7.9 nm (referenced to “ready‐to‐use” transducer), with the LSPR curve peaking at 419.67 nm. The comparison of UV–VIS spectra for positive control in Figure 2a,b illustrates the importance of the specific Ab. If the positive control (polyclonal antibody) is interacting with the bare AgNPs/C:H:N:O nanocomposite with C:H:N:O overcoat (“buffer + block + positive ctrl” curve in Figure 2a, the LSPR shift is observed at about 3.3 nm, while in case of the specific antibody, the LSPR shift is more than 2 times larger (Δλ_LSPR_ = 7.9 nm), see “specific Ab + block + positive ctrl”). This difference indicates that the specific Ab—positive control interaction is more dominant than the interaction of surface —NH_2_ with positive control grafting. At this point, the possible effect of the size of used specific and rabbit anti‐dolphin polyclonal antibodies (10–15 nm) is also worth noting. The positive control is grafted through the specific antibody, which enlarges the distance between the detected positive control (polyclonal antibodies) agents from the LSPR active surface and subsequently reduces the LSPR shift.

To support these results for the specificity of the LSPR shift of the “ready‐to‐use” sensor, immunofluorescence was performed to visualize the attachment of specific antibodies (anti‐DbPA) in comparison to non‐specific antibodies. Figure 2c shows the strong fluorescence signal in the “ready‐to‐use” sensor. On the other hand, when the non‐specific antibody (non‐immunized serum) is used, the signal is weak. The weak signal is caused by antibodies that are normally present in all non‐immunized areas since the immune system produces the normal level of antibodies that can also attach to our surface in a small ratio. To support the immunofluorescence results, the concentration of the attached specific antibody was measured. ELISA confirmed that 81.52 ng/mL of specific antibody was attached to the sensor transducer area in comparison to 41.52 μg/mL of non‐specific antibody.

### 3.2 Selective Immobilization and Detection of Lyme Disease Pathogen

**FIGURE 3:**
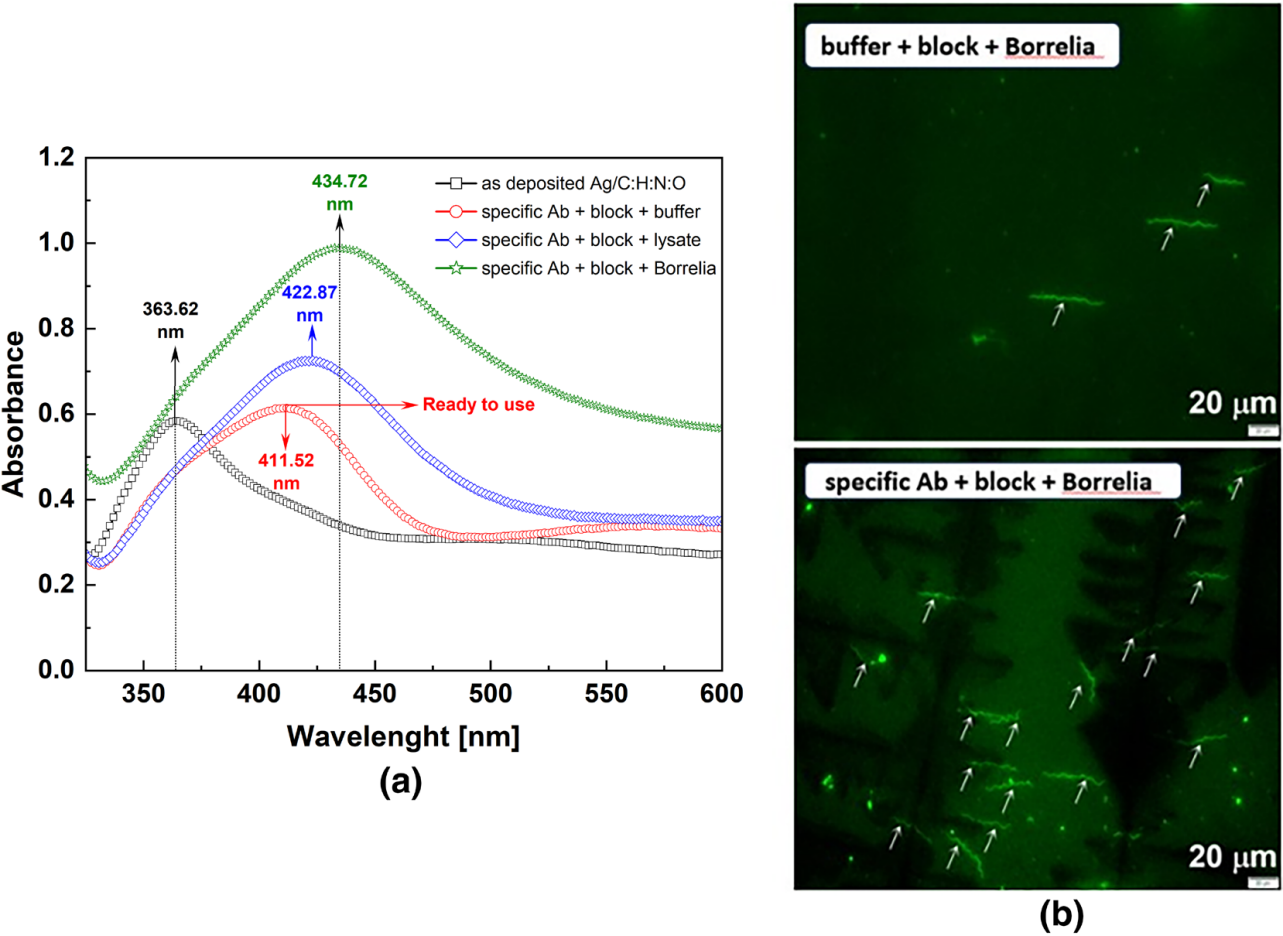
(a) LSPR modulation of UV–VIS spectra by the selective immobilization of Borrelia pathogens: (i) as‐deposited AgNPs/C:H:N:O with λ_1_ = 363.62 nm, (ii) surface with 10 nm C:H:N:O, primary antibodies and stabilized by blocking buffer with λ_2_ = 411.52 nm so‐called “ready‐to‐use” transducer, (iii) Borrelia protein lysate λ_3_ = 422.87 nm, and (iv) LSPR absorption during the detection of live Borrelia spirochetes, λ_4_ = 434.72 nm. (b) Detection of attached Borrelia by fluorescence microscopy. A concentration of 2.7⋅10^7^ Borrelia per sample with a detection area of 0.785 cm^2^ was used.

The modulation of the LSPR after the immobilization of Borrelia protein lysate and live intact Borrelia on the functionalized surface, respectively, is shown in Figure 3a. Both are captured by the specific antibody in the form of Anti‐DbpA serum. There are four characteristic LSPR curves presented in Figure 3a: (i) the LSPR marked “as‐deposited” AgNPs/C:H:N:O nanocomposite, (ii) the LSPR curve “specific Ab + block + buffer” representing functionalized surface by the plasma polymer and specific antibody, i.e., so‐called “ready‐to‐use” transducer, (iii) the LSPR modulated by the immobilization of the Borrelia protein lysate termed as “specific Ab + block + lysate”, and (iv) the LSPR curve modulated by the grafting of the live *Borrelia afzelii strain CB43* that is denoted as “specific Ab + block + Borrelia”. The LSPR peaks related to the surface (i) and (ii) are 363.62 nm and 411.52 nm, respectively, which slightly differs from those “identical surfaces” shown in Figure 2a,b. The reason is the error caused by the preparation of the samples of different production batches.

The “ready‐to‐use” transducer with the functionalized surface is considered here as a reference with λ_LSPR_ = 411.52 nm. In the case of attachment of the Borrelia protein lysate by the specific antibody, the LSPR shift Δλ_LSPR_ = 11.35 nm and a rise of the absorbance of about 45% are observed, see Figure 3a. Here, the key parameters responsible for the LSPR modulation are the size of the marker protein and the density of immobilized proteins ∑_PROT_, i.e., the number of immobilized proteins per surface area unit. Even more significant LSPR shift (Δλ_LSPR_ = 23.2 nm) and higher absorption enhancement were caused by the attachment of Borrelia spirochetes, indicating the high sensitivity of the detection system. The more pronounced shift of the LSPR peak, caused by live Borrelia compared to the detected Borrelia lysate, is due to the significantly larger Borrelia size compared to individual proteins; Borrelia, typically 20–30 μm long and 0.2–0.3 μm wide while the protein size is ~5–10 nm.

The immobilization (capture) of live Borrelia onto the surfaces was verified by fluorescence microscopy (FM). Figure 3b shows the attachment of Borrelia on the sensor surface compared to the control sample. Borrelia, which can be recognized by its typical spiral shape in Figure 3b, seems to be attached to a single point being oriented in the vertical position concerning the substrate. In such a case, it becomes difficult to detect Borrelia clearly or even at all, because of variable microscope focal length. In addition, Borrelia were attached in grouped islands, making the quantification of their number on a surface impossible. In the FM image of a control sample in Figure 3b (buffer + block + Borrelia), a few spirochetes are visibly attached to an unfunctionalized surface; such binding is expected and mostly present irrespective of the surface used being a natural error of detection. Bacterial membranes are coated by various types of adhesions providing specific and non‐specific binding to various surfaces [69, 70, 71, 72]. Blocking steps used were selected to limit this non‐specific binding to a minimum, which is apparent from Figure 3b where the higher surface density of Borrelia immobilized by the specific Ab as compared with the control sample is visible.

**FIGURE 4:**
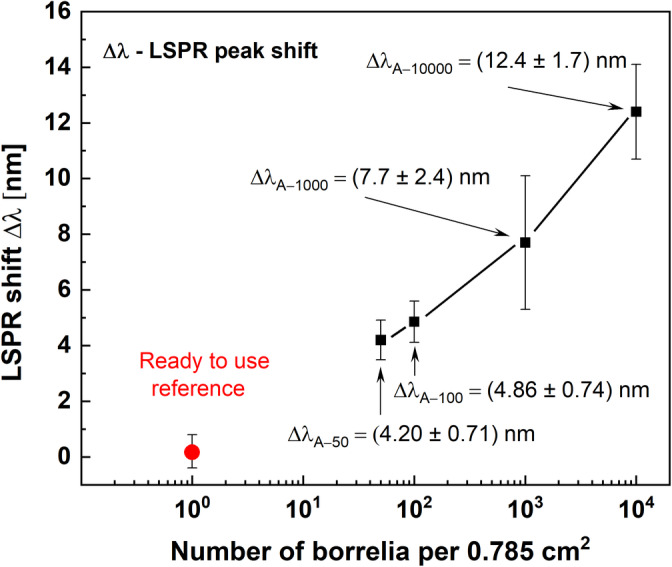
LSPR shift for different numbers of Borrelias inoculated on a detection area of 0.785 cm^2^. The LSPR red‐shift is caused by Borrelia immobilized onto the transducer surface with a detection limit of 50 Borrelias per sample, corresponding with LSPR shift Δλ_50_ ≈ (4.20 ± 0.71) nm.

To verify the sensitivity of the sensor transducer, different concentrations of Borrelia, their immobilization ability, and their LSPR response were tested. In this investigation, the sample was represented by a glass substrate, 10 mm in diameter with an effective area A = 0.785 cm^2^, that was incubated with the cultivation media containing different numbers of Borrelia (50, 100, 1000, and 10 000 Borrelia/sample). The measured LSPR absorption curves and corresponding LSPR shifts are presented in Figure 4. The AgNPs/C:H:N:O nanocomposite, functionalized by both C:H:N:O film and primary Ab, i.e. a “ready‐to‐use” transducer serves as a reference. For the highest number of 10 000 Borrelia, the LSPR red‐shift Δλ_A‐10000_ = (12.4 ± 1.7) nm was measured. Furthermore, the LSPR shift decreases with decreasing number of Borrelia in the medium tested: Δλ_A‐1000_ = (7.7 ± 2.4) nm, Δλ_A‐100_ = (4.86 ± 0.74) nm, and Δλ_A‐50_ = (4.20 ± 0.71) nm, respectively (Figure 4). This proves that the presented LSPR‐based strategy for Borrelia detection is sensitive enough to identify their presence down to several tens of Borrelia on the sensor area of 0.785 cm^2^.

However, it is important to note that we experimented with 10 Borrelia, but the LSPR shift for this amount is as low as 1 nm, i.e., the value interfering with measurement and processing errors, namely, if treated in praxis. The importance of the stated limit of detection is the order of magnitude, which means 50 of Borrelia detected and, in addition, this level of detection limit is achieved without any highly complex or destructive way of sample preparation or extraction. Such a limit of detection is comparable with commercially available diagnostic IVD kits currently in the market, but more importantly with a less complicated and quicker analysis. Hence, the practical detection limit of the developed transducer is in the range of 50 Borrelia, which caused the LSPR shift Δλ > (4.20 ± 0.71) nm, which is a value measurable using conventional UV–VIS spectrometers.

## 4 Conclusions

We demonstrated the ability of functionalized AgNPs/C:H:N:O nanocomposite to act as a selective and sensitive transducer for the detection of live *B. afzelii* spirochetes. The immobilization of Borrelia, either live or as lysate, is indicated by the modulation of the LSPR signal due to optical and electrical changes in the closest surface environment. Live spirochetes were immobilized by the Anti‐DbpA serum and well‐anchored onto the surface by —NH_2_ grafts of plasma polymerized nylon. We proved that the transducer detection limit ranges in order 50 Borrelia per area of 0.785 cm^2^ when the LSPR shifts Δλ > (4.20 ± 0.71) nm. Such shift is detectable by absorbance measurement utilizing a conventional UV–VIS spectrometer. This level of sensitivity for live spirochetes is high and compares well with diagnostic kits available either commercially or in‐house [73, 74, 75] or known as proof of concept [76]. The benefit of the introduced transducer strategy lies in easy preparation and low‐cost production [58, 59] and friendly practical usage suitable for potential quick and specific point‐of‐care testing not only for Lyme Disease but for diseases caused by other pathogenic microorganisms.

## Conflicts of Interest

The authors declare no conflicts of interest.

## Supporting information


Data S1.

## Data Availability

The data that support the findings of this study are available from the corresponding author upon reasonable request.
