# Supplementary material for: Tailored Functionalization of Plasmonic AgNPs/C:H:N:O Nanocomposite for Sensitive and Selective Detection
Source: J Biophotonics. 2024 Dec 23;18(2):e202400353. doi: 10.1002/jbio.202400353 (PMC11793947; doi:10.1002/jbio.202400353)
Supplement: Supplementary file 1 — Data S1. [file JBIO-18-e202400353-s001.docx]

Supporting Information

**Tailored functionalization of plasmonic Ag/C:H:N:O nanocomposite for sensitive and selective detection**

Sanjay Kumar^1*^, Hana Maskova^1,2^, Anna Kuzminova^3^, Paval Curda^1^, Lenka Doudova^1,2^, Jan Sterba^1^, Ondřej Kylián^3^, Ryan O. M. Rego^1,2^, Vitezslav Stranak^1^

^1^University of South Bohemia, Faculty of Science, Branisovska 1760, 37005, Ceske Budejovice, Czech Republic

^2^Biology Centre ASCR, Institute of Parasitology, Branisovska 31, 37005 Ceske Budejovice, Czech Republic

^3^Charles University, Faculty of Mathematics and Physics, V Holešovičkách 2, 180 00, Prague 8, Czech Republic

*Correspondence Email: [kumars00@prf.jcu.cz](mailto:kumars00@prf.jcu.cz)

**Table S1.** Control measurements to verify the surface functionalization and origin of the LSPR modulation.

| **Sr. No.** | **Type of control** | **Solutions/Specimens used for detection** |
| --- | --- | --- |
| (1) | buffer + buffer + buffer | PBS-T + TBS-T + TBS |
| (2) | buffer + block + buffer | PBS-T + 0.15 M Glycine in TBS-T + TBS |
| (3) | specific Ab + block + buffer | Primary Ab in PBS-T + 0.15 M glycine in TBS-T + TBS |
| (4) | buffer + block +  positive control (Lysate/Borrelia) | PBS-T + 0.15 M Glycine in TBS-T +  polyclonal Ab/Lysate/Live Borrelia in TBS |

**Table S2.:** Detection of specimens through AgNPs/C:H:N:O nanocomposite and the LSPR response.

| **Sr. No.** | **Combination used for detection** | **Specimens used for detection** |
| --- | --- | --- |
| (a) | specific Ab + block + positive control | goat anti-rabbit HRP antibody in PBS-T (1:1000) + 0.15 M Glycine in TBS-T + rabbit anti-dolphin polyclonal antibody |
| (b) | specific Ab + block + Lysate | Anti-DbpA serum in PBS-T (1:200) + 0.15 M Glycine in TBS-T + Borrelia Lysate in TBS |
| (c) | specific Ab + block + Borrelia | Anti-DbpA serum in PBS-T (1:200) + 0.15 M Glycine in TBS-T + Borrelia in TBS |

**S3:** Determination of the antiDbPA antibody concentration.

Rabbit OASL antibody (PA5-81946, Invitrogen) was used to create a standard calibration curve since the exact concentration is known. The rabbit anti-DbPA antibody was diluted similarly as in experiments, 1:200 in PBS. The standard antibody and anti-DbPA antibody (100 μl) were bound onto HighBond ELISA plates for 1 hour at 37 °C. PBS was used as a blank. After this period, three washing steps in PBS-T were performed. Non-specific bonds were blocked using 4% BSA in PBS (200 μl) for 45 min at 37 °C. Three washing steps in PBS-T were then carried out. HRP Goat Anti-Rabbit IgG (H+L) Antibody (Peroxidase) (AS014, Abclonal; 100 μl) was added to each well (1:2500) and incubated for 1 hour at 37 °C. After the PBS-T washing steps were performed, 100 μl of TMB substrate (ES022, Merck) was added. Wells were incubated for one minute at 37°C. Reactions were stopped using 0.3 M sulphuric acid. Absorbances were measured by Synergy H1 microplate reader (Biotek) at 450 nm. Blank was subtracted from all measured samples. According to the equation, the concentration of specific antibody stock was calculated, as well as the concentration of the attached antibody on the sensor transducer area.

***Figure S4****. Standard curve for the determination of the specific antibody concentration of stock and the concentration of attached specific antibody. The standard curve was created using MS Office Excel.*
